# Ecological Network Indicators of Ecosystem Status and Change in the Baltic Sea

**DOI:** 10.1371/journal.pone.0075439

**Published:** 2013-10-07

**Authors:** Maciej T. Tomczak, Johanna J. Heymans, Johanna Yletyinen, Susa Niiranen, Saskia A. Otto, Thorsten Blenckner

**Affiliations:** 1 Baltic Sea Centre, Stockholm University, Stockholm, Sweden; 2 Scottish Association for Marine Science, Scottish Marine Institute, Dunbeg, Oban, United Kingdom; 3 Nordic Centre for Research on Marine Ecosystems and Resources under Climate Change (NorMER), Stockholm Resilience Centre, Stockholm University, Stockholm, Sweden; 4 Stockholm Resilience Centre, Stockholm University, Stockholm, Sweden; Technical University of Denmark, Denmark

## Abstract

Several marine ecosystems under anthropogenic pressure have experienced shifts from one ecological state to another. In the central Baltic Sea, the regime shift of the 1980s has been associated with food-web reorganization and redirection of energy flow pathways. These long-term dynamics from 1974 to 2006 have been simulated here using a food-web model forced by climate and fishing. Ecological network analysis was performed to calculate indices of ecosystem change. The model replicated the regime shift. The analyses of indicators suggested that the system’s resilience was higher prior to 1988 and lower thereafter. The ecosystem topology also changed from a web-like structure to a linearized food-web.

## Introduction

Many marine ecosystems are under pressure due to multiple drivers, such as fishing, climate change and eutrophication, causing large-scale food-web reorganizations, often called regime shifts [1]. The definitions of regime shifts vary, see for example Lees *et al*. [1]. In this study, we use the definition of McKinnell *et al.* [2] who state that: “regime shifts are low-frequency, high-amplitude and sometimes abrupt changes in species abundance, community composition and trophic organization that occur concurrently with physical changes in the climate system”.

McKinnell *et al*. [2] and Cury and Shannon [3], [4] highlight changes of the internal structure, organization and size of an ecosystem as characteristic of regime shifts. Regime shifts have been described in several marine ecosystems such as the Southern and Northern Benguela [3], [5], [6], Southeast Alaska and Aleutian Islands [7] and the Black Sea [8]. All of these regime shifts have the re-organisation of food-webs in common. In general, food-web re-organizations are best described by Ecological Network Analysis (ENA) *sensu* Ulanowicz [9]. The network approach to ecological research provides a powerful representation of the pattern of interactions among species; highlights their interdependence and equips ecologists to find generalities among seemingly different systems [10]. Knowledge of the network topology (e.g. connectance, number of species, interaction rates) provides insights to ecosystem functioning and stability, highlights the advantages of integrating network research with empirical indicators of resilience, and uncovers generic features of these complex systems [10]–[16].

**Figure 1:**
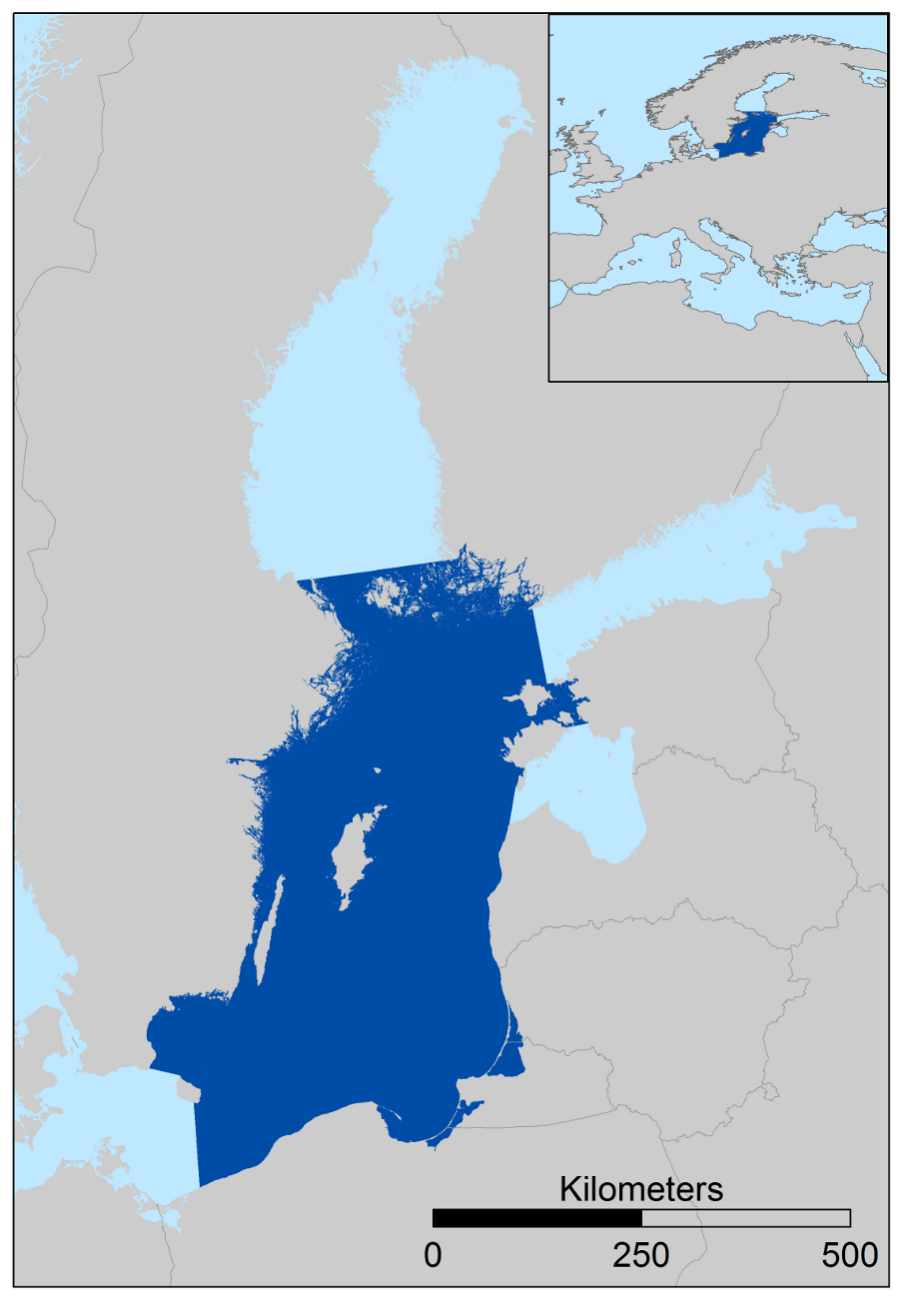
The Baltic Sea with the study area, the Baltic Proper (dark).

In the Baltic Sea (Fig. 1), an ecosystem regime shift has been described for the Central Basin (Baltic Proper) in the late 1980s [17], [18]. This regime shift included pronounced changes and reorganizations within and across the trophic levels of zooplankton and fish [17], [18]. Network analyses have already been applied to the Baltic Sea. For example, Wulff *et al*. [19] used the ENA method to compare the Baltic Sea to Chesapeake Bay and Tomczak *et al*. [20] used ENA to compare coastal ecosystem maturation and stress in five coastal ecosystems. However, none of these Baltic related ENA studies took temporal changes in the ENA indices and regime shifts into account. Similarly, a number of studies have analysed food-web changes in other marine systems using ENA indices [4], [5], [21]–[23], but very few have used a time dynamic approach [7].

Thus, we focused on changes in the resilience of the Baltic ecosystem to describe and understand the processes underlying the regime shift. We investigated food-web reorganisation at the ecosystem level as revealed by the theory of ecological succession and maturity [9], [24], [25], which is linked to the theory of resilience [26] and regime shifts [27], [28]. Therefore, the aim of this study was to calculate temporally integrated ENA and ecological indices to test for dynamic changes in the food-web in relation to the suggested regime shift, and to explain these changes in relation to the resilience and trophodynamic properties of the Baltic Sea ecosystem.

## Materials and Methods

### Study Site

The Baltic Sea hydrographical conditions are characterized by: (i) a horizontal sea surface salinity gradient from 10 PSU in the South-West to 6 PSU in the North-Eastern part of the Baltic Proper [29], (ii) high riverine inflows [30], and (iii) major, irregular, inflows of saline, oxygenated water from the North Sea leading to a permanent pycnocline that partly contributes to deep-water hypoxia [31], [32]. During the last century, high land-based nutrient loads have led to the eutrophication of the Baltic Sea with typical eutrophication-related symptoms, such as massive cyanobacteria blooms in summer and widespread deep-water anoxia [31], [33].

Fisheries have heavily exploited the Baltic Sea resources [34]. Landings of the main commercial fish stock, Eastern Baltic cod (*Gadus morhua calarias*), increased dramatically at the beginning of the 1980s and collapsed in the early 1990s [35]. During that time cod biomass has declined severely [36]. Instead, small pelagic fish, such as sprat (*Sprattus sprattus*) and herring (*Clupea harengus*), have dominated the catches during the last 20 years [35]. Möllmann *et al*. [18] suggested that high fishing pressure on cod contributed to its decline, and the resulting trophic effects cascaded down to the copepods (*Pseudocalanus acuspes).* Increasing temperature positively affected zooplankton (*Acartia* spp.) abundance, sprat reproduction, and consequently established the current regime of *Acartia* spp. and sprat dominance [37].

### Modelling Approach and Model Description

Ecopath with Ecosim [38] was created for building food-web models (www.ecopath.org). The dynamic extension of Ecopath that allows temporal analysis and fitting the model to time series is undertaken by Ecosim, using the master equation (1)
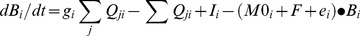
(1)where dB_i_/dt represents the growth rate during the time interval dt of group (i) in terms of its biomass (B_i_), g_i_ is the net growth efficiency (production/consumption ratio), Q_ji_ is the consumption rates, M0_i_ the non-predation (‘other’) natural mortality rate, F_i_ is fishing mortality rate, e_i_ is emigration rate, I_i_ is immigration rate (and e_i*_B_i_-I_i_ is the net migration rate).

**Figure 2:**
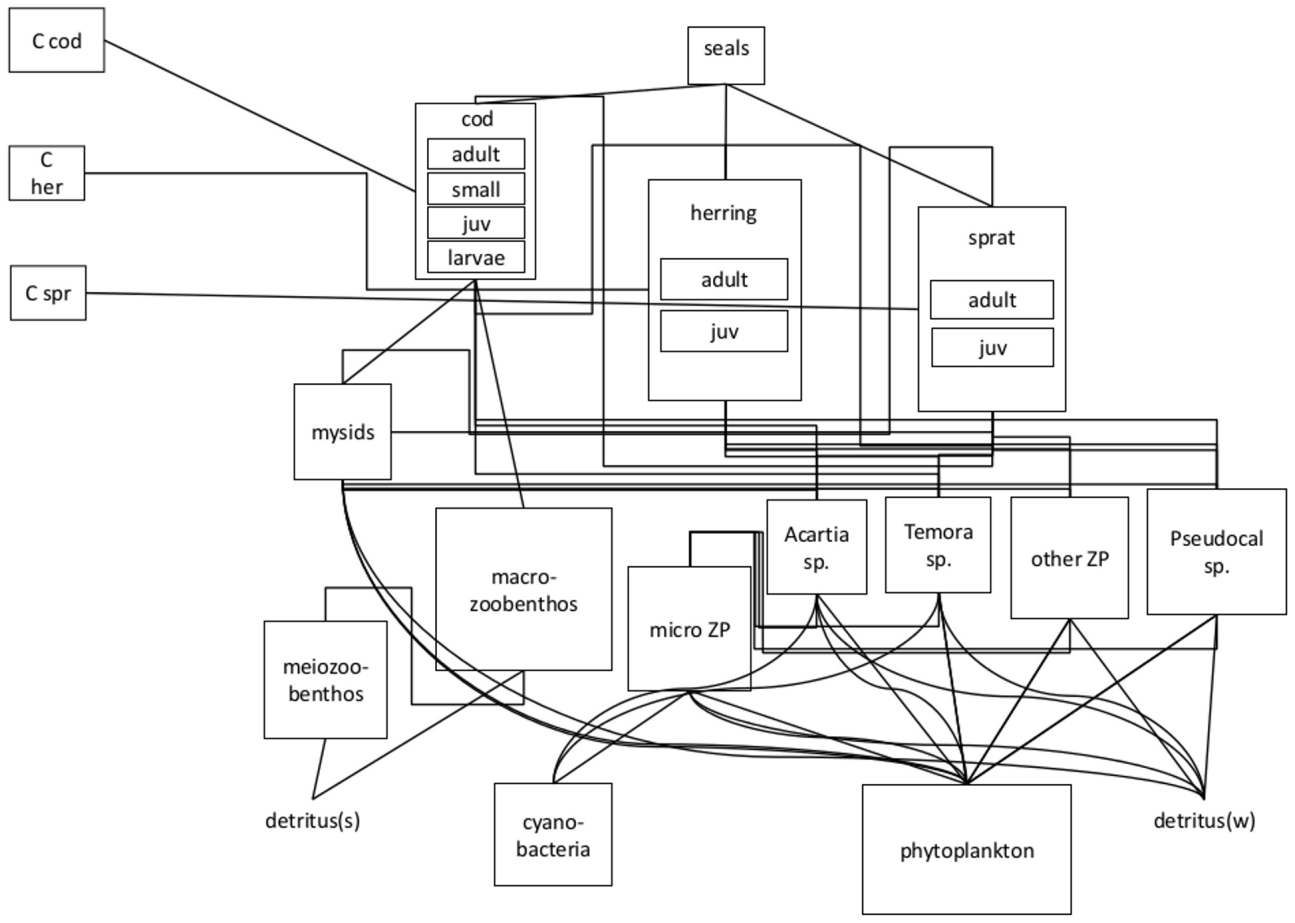
The structure of the food-web model, also indicating the fishing pressure (F) for the respective fisheries on the three fish species, pp – primary producers, juv – juvenile stanza of given fish species. Detritus pool is divided into two groups: detritus on the sediment (detritus (s)) and water column detritus (detritus (w)).

The current Baltic Ecopath with Ecosim model, based on Tomczak *et al*. [39], covers the area of the Central Baltic Sea (ICES subdivisions 25–29, excluding Gulf of Riga) and contains 21 functional groups (Fig. 2), including three fishing fleets on the main commercial fish species: cod, sprat and herring. For details see Tab. S1–S3 and Fig. S1–S3 in File S1.

### Data and Analysis

Three analyses were performed to: i) test for abrupt changes in the observed data that were used to force the ecosystem model, ii) test for abrupt changes in the modelled biomass, and iii) analyse ecosystem properties (ENA indices) in reference to observed changes [18], and the ecological theories of Scheffer and Carpenter [28], Folke *et al.* [40] and Odum [24] (see section Linking theory).

### Forcing Data and Simulated Biomass

The forcing data represent both environmental and human impacts on the Baltic Sea food-web (Fig. S2 in File S1). Temporal anomalies of sea surface temperature in August and the spring temperature from 0–50 m depth (SST_aug; TempWC_spring), primary production (PP_BALTSEM), hypoxic area, Cod Reproductive Volume (CodRV [41]), herring recruitment (HER_rec), as well as fishing on small and adult cod (FSmallCod, FAdCod), sprat (FJuvSprat, FAdSprat) and herring (FJuvHerr, FAdHerr) were analysed to test for non-linear shifts. For the time series data used see references and application details in Tomczak *et al.* [39]. The modelled biomasses of 19 of the 21 functional groups were included in the statistical analysis (see section Statistical analysis and Fig. S3 in File S1). Seals and detritus were excluded from the dataset to ensure that the data were comparable and consistent with the number of trophic levels used in Möllmann *et al*. [18]. A further motivation for exclusion of the seal and detritus data was that seal biomass was used as one of the forcings in the model, while detritus showed very high cross-correlation with PP.

### Ecosystem Indicators and Ecological Network Analysis Indices

**Figure 3:**
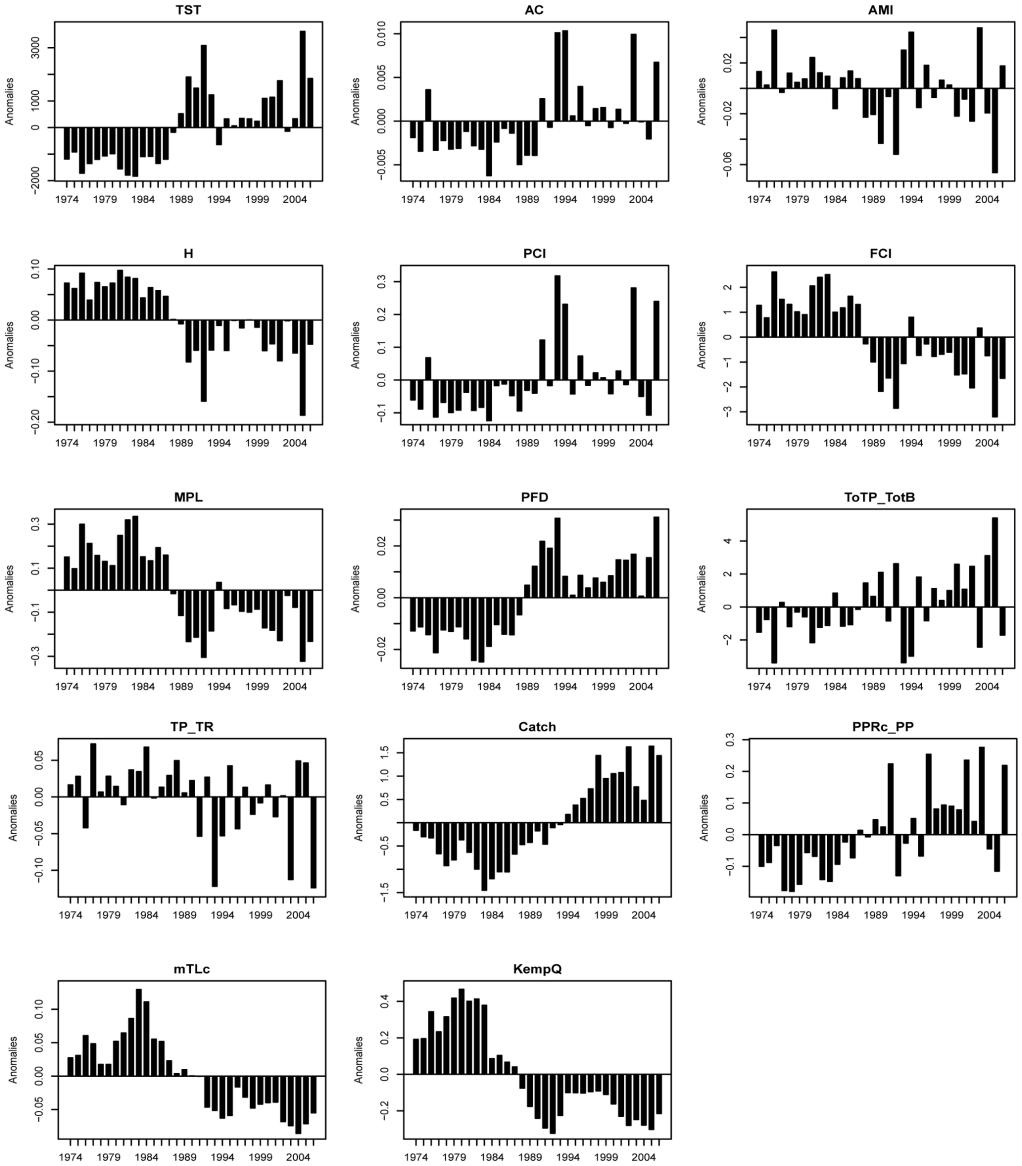
Ecological indicators and ENA indices anomalies (note different scale) from 1974–2006.

We calculated 15 network analysis indices, ecosystem metrics and biomass diversity indices (Fig. 3; for definitions and descriptions see Tab. S5 in File S1), commonly used to describe changes in ecosystem properties and food-web dynamics [7], [9], [19], [21], [22], [24], [25], [42]–[54]. Indices were assigned to a number of groups, describing ecosystem properties in terms of ecosystem/food-web resilience and structure, and fisheries. Structure indices included: Total System Throughput (*TST*), Relative Ascendancy (*A/C*), Redundancy (*R*), Average Mutual Information (*AMI*), Entropy (*H*), Mean Path Length (*MPL*), Kempton-Q index (*Q*), recycling within the ecosystem: Finn Cycle Index (*FCI*), Predatory Cycle Index (*PCI*), Proportional Flow to Detritus (*PFD*), System turnover rate (*ToTP/ToTB*), and Total Primary Production per Total Respiration (*TPP/TR*). Fisheries impact indices included: Primary Production Required to sustain catch per Primary Production (*PPR/PP*), mean Trophic Level of catch (*mTLc*), and Total Catch (*Tot C*).

### Linking Theory

Mageau *et al.* [25] used ENA indices to define system health [55] and maturation [43] and concluded that an unstressed (healthy) ecosystem is able to maintain its structure (organization) and function (vigor) over time in the face of external stress (resilience). Vigor is a measure of system activity, metabolism or production [55]. Organization is a measure of the number and diversity of interactions between the components of a system, and resilience refers to the ability of a system to maintain its structure and function in the presence of stress [25]. Odum [56] and Ulanowicz [9] suggested that stressed ecosystems are characterized by an inhibition or even reversal of the trends associated with ecosystem development. In this paper we specifically refer to proxies of resilience - namely redundancy (Tab. S5 in File S1), linked by Christensen [43] with system stability and proposed by Heymans *et al.* [7] as an index of food-web resilience. According to Bondavalli *et al.* [57] high redundancy signifies that either the system is maintaining a higher number of parallel trophic channels in order to compensate for the effects of environmental stress, or that it is well along its way to maturity. At the same time, Scheffer *et al.* [27] and Scheffer and Carpenter [28] defined resilience as the “depth of the basin of attraction”. We link resilience to changes in redundancy, by assuming that *R* is a proxy of resilience as given by Christensen [43] and Heymans *et al*. [7].

### Statistical Analysis

A series of statistical methods (see sections below) as described in Diekmann and Möllmann [58] were applied to the time series of model forcing (force) and modelled biomass (mb): 1) Sequential *t*-test analyses of regime shifts (STARS) [59]; 2) Principal Component Analysis (PCA); 3) STARS on PCA scores and 4) Chronological Clustering Analysis (CC) [60]. Due to high cross-correlations (see Tab. S6 in File S1), we did not perform a PCA on the ENA indices. Instead, ENA indices were analysed using STARS and integrated using CC. For ENA indices, coefficient of variation (CV) was estimated to examine the variability in the given time periods. A traffic light plot was used to visualise the dynamics of subsequent data sets (forcing and biomass).

### Sequential t-test Analyses of Regime Shifts (STARS)

To recognize significant shifts in mean values of a given time series, a sequential *t*-test on the mean (STARS) was applied for each time series separately. The two parameters that control the scale and magnitude of potential regime shifts were set *a priori*. The significance level (α) was set to 0.05. The cut-off length (*l*) was set to five for forcing variables and indices, to test for changes in “fast” environmental variables and examine specific periods of changes between regimes. For modelled biomasses, the cut-off length was set to 10 years for comparison to Möllmann *et al.* [18]. The calculation of shifts was also affected by the handling of outliers. Thus, the Huber’s weight parameter (which controls the identification and weights assigned to outliers [59]) was set to 3. Therefore, if the deviation of a measurement from the expected average (normalized by its standard deviation) was >3, its weight was inversely proportional to the distance from the expected mean value. Shifts detected in the very last years were not taken into account during the analysis due to the known limitation of this method [61].

### Principal Component Analysis (PCA)

Standardized PCA, based on the correlation matrix, was carried out on the transformed values (ln+1) of the given data set. First, a PCA was applied for forcing (force) and modelled biomass (mb) time series. The PC1 scores on the forcing variables (PC1_force_) were used as an index of pressure and the scores of the modelled biomass were used as an index of biological change (PC1_mb_). Annual scores of the two principal components, PC1_mb_ and PC2_mb_, were plotted against time to visualise temporal relationships and the occurrence of abrupt modelled system changes. Variable loadings and scores were displayed on the 1^st^ factorial plane, and the years were chronologically connected to show the pressure/state trajectory [18].

### STARS on PCA Index Time Series

STARS were used to detect sudden changes in the PC scores to identify whether abrupt changes had occurred [18]. Parameters for the analyses were set as described in sections above.

### Chronological Clustering (CC)

Independently of the STARS and PCA analyses, a second discontinuity analysis, CC, was carried out to identify the years in which the largest shifts in the mean value of the time series occurred. This method groups sequential years based on a time-variable matrix [61]. To demonstrate the most important breakpoints in the dataset, the significance level (α), which can be considered as a clustering-intensity parameter, was set to 0.01. The connectedness level was set to 50%. In accordance with the use of the correlation coefficient in the PCA, the data were first normalized, and then the Euclidean distance was calculated to determine similarity between years.

### Traffic Light Plots (TLP)

To visualise overall systematic patterns based on single time series, TLPs were generated [62]. The modelled biomass values of each functional group were categorized into quintiles and each quantile was assigned a specific colour: green for the lowest (0–20%), red for the highest (80–100%) and a gradation of colours in between. The variables were then sorted in descending order according to their PC1 loadings, and plotted against years to visualise temporal patterns.

## Results

### Changes in External Forcing

**Figure 4:**
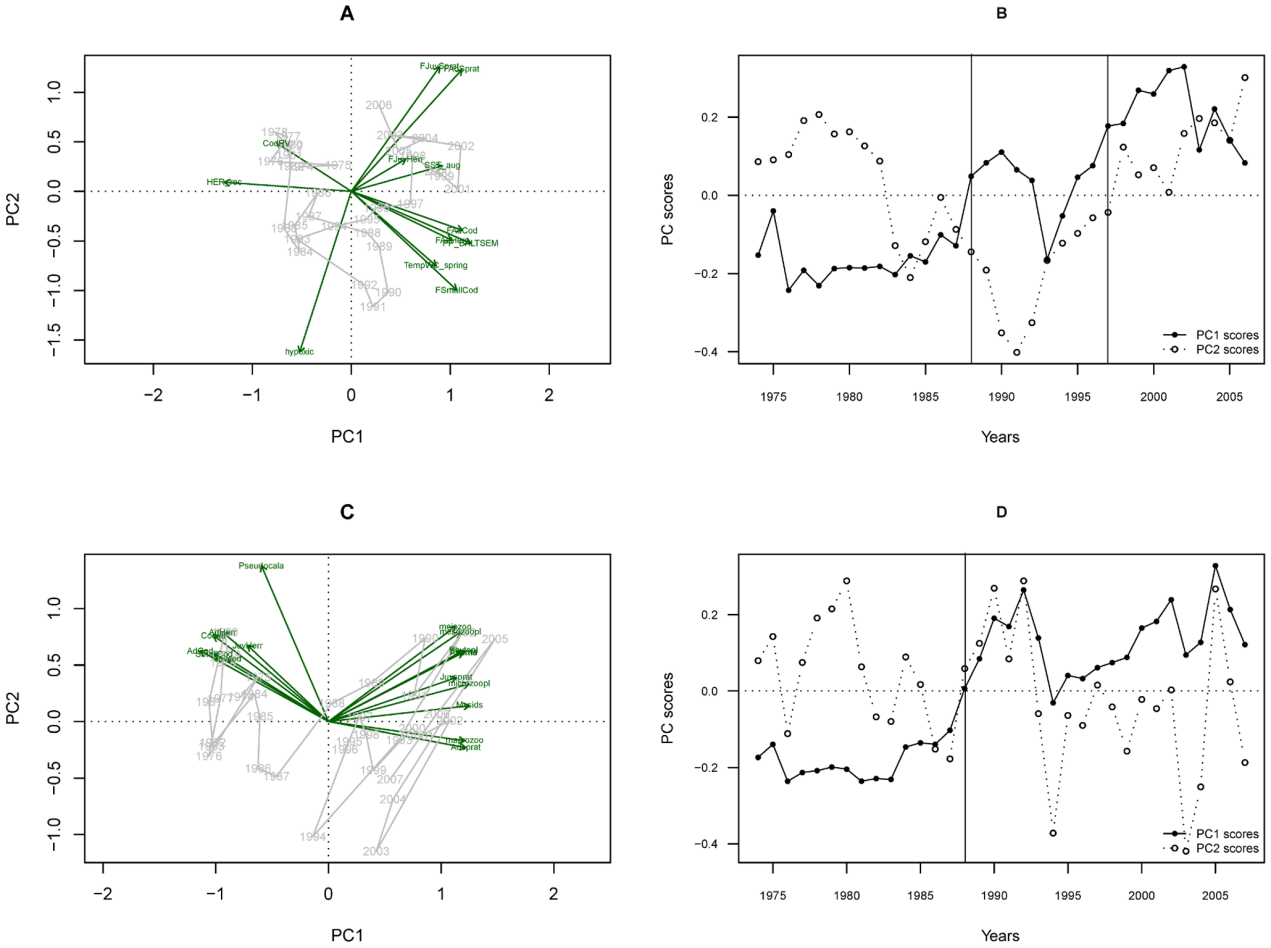
Results of the Principal Component Analyses, with the first and second principal component (PC1 and PC2). The first column shows the dependencies between variable – (A and C) (for detailed values see Tab. S4 in File S1) the second column shows temporal trend PC1 and PC2 axis scores (B and D). Rows show the results of analyses of data sets including: model forcing (A and B), and modeled biomass (C and D), respectively. Vertical lines on PC components time trajectory represents shifts tested by the regime shift analysis. Please note that the scale differs between axes.

**Figure 5:**
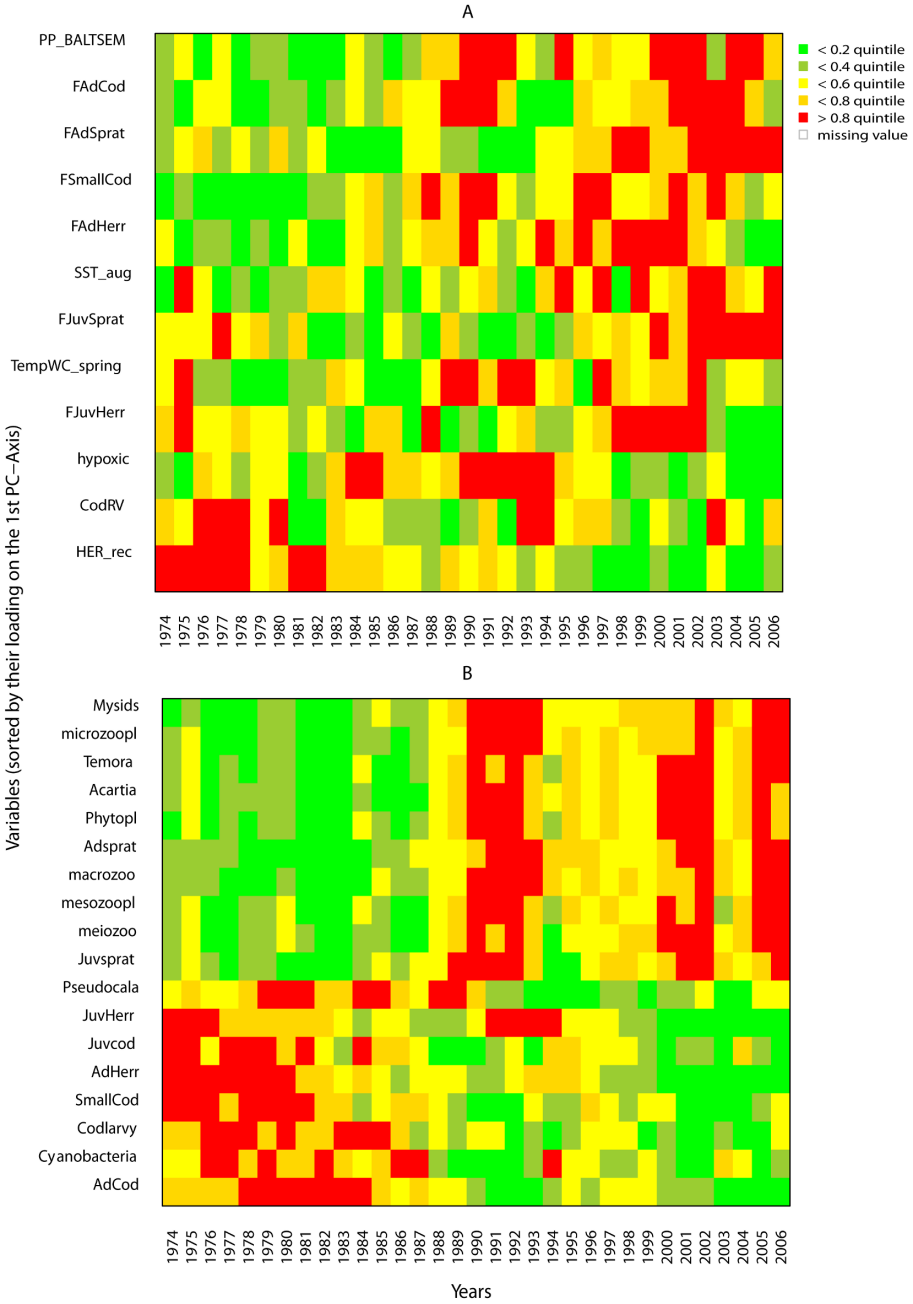
Traffic light plots representing the applied forcing and development of the simulated biomass from the different groups. The time series were transformed into quintiles and sorted according to the PC1 axis scores: (A) model forcing; (B) modeled biomass.

Many of the observed changes in model forcing time series (Tab. 1) occur in the mid-late 1980s and mid-late 1990s, with some shifts appearing after 2000 (Fishing mortality of Juv. Sprat, Juv. Herring and Adult Herring). PCA on the forcing time series (Fig. 4) indicates a strong change in the overall pressure on the ecosystem (Fig. 4A) with changes in PC1_force_ and PC2_force_ indices (Fig. 4B) explaining 33% and 18% of the total variation, respectively. Forcing variables that contributed most to PC1_force_ were: PP, Cod RV, herring recruitment and the fishing mortality on cod and sprat (Fig. 4A, Tab. S4 in File S1). The shifts in the forcing index (i.e., PC1_force_) occurred in 1988 and 1997 (Fig. 4B, Tab. 2) while CC detected data discontinuity at 1983, 1998 and 2003 (Tab. 3). The traffic light plot (Fig. 5) illustrates the temporal changes in forcing factors as well as modelled biomass: Fig. 5A shows differences before (low PP, high Cod RV and low fishing) and after the mid-1980s regime shift (with high PP suggesting eutrophication, high fishing, increased temperature and unfavourable cod reproduction conditions).

**Table 1:** Timing of shifts detected using STARS, given time series of model forcing variables.

Forcing	Shift 1980s	Shift 1990s	Shift 2000s-
PP	1989		2005
SST_August			2006
Temp 0_50m_spring	1989		2006
CodRV	1981		
Hypoxic_Area	1983	1998	
HER_rec	1986		
F_JuvSprat		2000	2006
F_AdSprat	1983	1994	
F_JuvHerr		1998	2003
F_AdHer		1994	2002
F_SmallCod	1987	1992	
F_AdCod	1989	1993;1999	2006

**Table 2:** Shifts in PC1 index detected by STARS.

Shifts at PC1	Shift 1980s	Shift 1990s	Shift 2000s-
Model Forcing	1988	1997	2006
Modeled Biomass	1988		2005

**Table 3:** Shifts in given data sets detected by Chronological Clustering.

CC alfa = 0.01	Shift 1980s	Shift 1990s	Shift 2000s-
Model Forcing	1983	1998	2003
Modeled Biomass	1988		
Modeled Indices	1989		

### Biomass State Change

The majority of significant shifts in modelled biomass occurred in the late 1980s (Tab. 4), with three time series showing additional shifts in the 1990s (*Pseudocalanus* sp., Juvenile Herring and Adult Herring). The first two axes of the PCA on the modelled biomasses (PC1_mb_ and PC2_mb_; Fig. 4C and D, Tab. S4 in File S1) explained 72% and respectively 10% of the total variance. The shift in biomass index (PC1_mb_, Fig. 4D) occurred in 1988, and is confirmed by the CC (Tab. 3). The PC_mb_ shifts (Tab. 2) and traffic light plot of modelled biomass (Fig. 5B) shows a clear dichotomy in the food-web, between cod vs. sprat and zooplankton vs. plankton (Fig. 5B).

**Table 4:** Timing of shifts detected using STARS, given time series of modeled biomass.

Modeled group	Shift 1980s	Shift 1990s	Shift 2000s-
Cyanobacteria	1988		2006
Phytoplankton	1988		
Microzooplankton	1988		
Temora sp.	1988		2005
Acartia sp.	1988		
Pseudocalanus sp.		1991	
Mesozooplankton	1988		2005
Mysids	1988		2005
Meiozoobenthos	1988		2005
Macrozoobenthos	1988		2005
Juvenile Sprat	1988		
Adult Sprat	1989		2006
Juvenile Herring		1994	
Adult Herring	1982	1997	
Cod larvae	1986		
Juvenile Cod	1982		
Small Cod	1984		
Adult Cod	1985		

### Emergent Food-web Changes

Similar to findings from the forcing and biomass time series, STARS detected shifts in most ecosystem descriptors and ENA indices at the end of the 1980s (1987/89) and the mid-1990s (1993/96) (Tab. 5). The ENA clearly shows two regimes, with a discrete step function between the end of the 1980s and the mid-1990s, described by Möllmann *et al.* [18] as a transitional period. No significant shifts were detected in *AMI*, turnover rate (*ToTP/ToTB*) or total primary production to respiration (*TPP/TR*), but the anomalies for almost all indicators fluctuated significantly (Fig. 3), showing extreme values between 1988 and 1995, and increased variability (higher CV) after the late 1980s (Tab. 6).

**Table 5:** Timing of shifts detected using STARS, given time series of indices.

Indices	Shift 1980s	Shift 1990s	Shift 2000s-
*TST*	1989	1994	2005
*A/C*		1993	
*R*	1989	1994	2005
*AMI*			
*H*	1988		2005
*FCI*	1988		2005
*PCI*		1993	2006
*MPL*	1988		2005
*PFD*	1989		2006
*ToTP/TotB*			
*TPP/TR*			2005
*Tot C*	1983; 1988	1995	2005
*PPR/PP*		1996	
*mTLc*	1982; 1987	1992	
*Kempton Q index*	1979;1984;1989	1994	2002

**Table 6:** Coefficient of variation of used indices for given time period (regime).

CV	1974–89	1990–2006	1974–2006
*TST*	0.108	0.148	0.215
*A/C*	0.007	0.014	0.014
*R*	0.013	0.017	0.031
*AMI*	0.012	0.022	0.018
*H*	0.006	0.011	0.015
*PCI*	0.160	0.310	0.318
*FCI*	0.097	0.144	0.188
*MPL*	0.035	0.033	0.061
*PFD*	0.014	0.017	0.030
*ToTP/TotB*	0.040	0.076	0.065
*TPP/TR*	0.022	0.045	0.039
*Tot C*	0.159	0.177	0.290
*PPR/PP*	0.183	0.244	0.285
*mTLc*	0.011	0.007	0.017
*Kempton Q index*	0.185	0.135	0.308

### Fisheries Affect Indicator Changes

Indicators directly related to exploitation reflect the shift in fisheries both in total yield and catch composition (Fig. 3 and Tab. 5). The first regime was characterized by high *mTLc*. The second regime had high total catch dominated by sprat, decreasing *mTLc* and increasing *PPR/PP* after the mid-1990s.

### Resilience and Regime Shift

**Figure 6:**
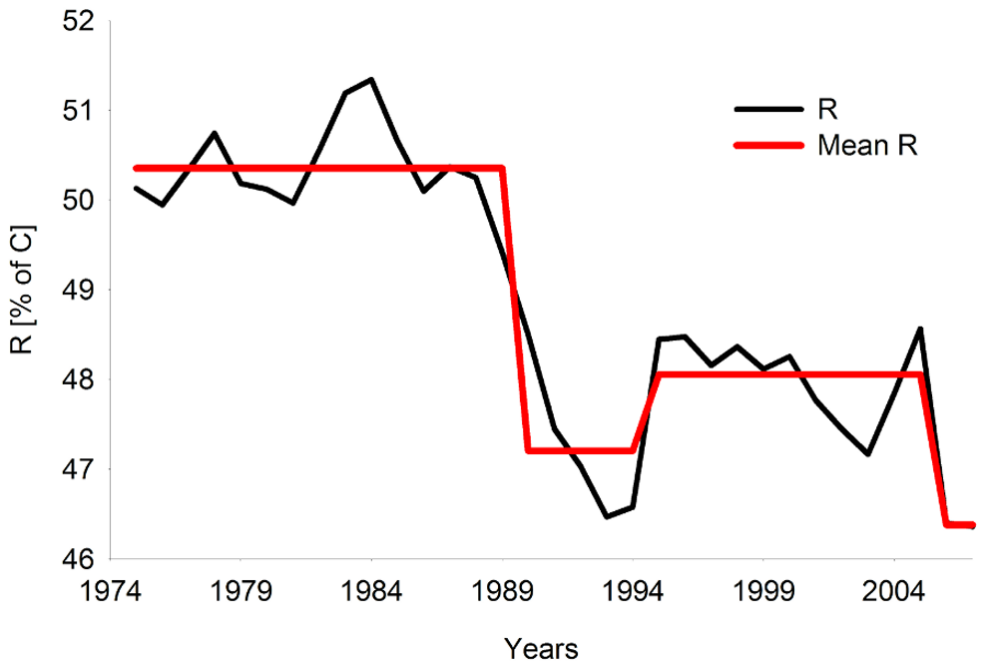
Time dynamics of redundancy (*R*) as percentage of capacity (*C*) in black and the red line represents the regime tested by the regime shift analysis for the period 1974–2006.

**Figure 7:**
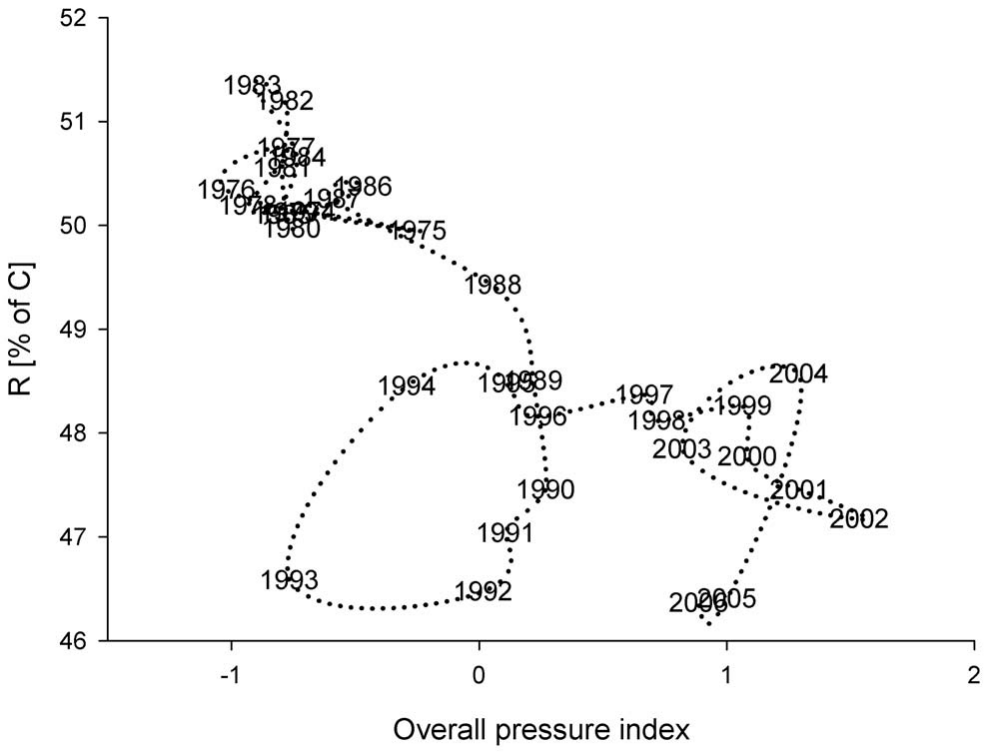
The redundancy (*R*) versus the overall pressure index, which is the principal component 1 from the model forcing variables.

The changes in redundancy (*R)* show a shift in 1989 and 1994 (Fig. 6). After 1994 a slight increase in *R* occurred, although not to the high values of the pre-1988 phase. The decrease in the *R* after the 1989 regime shift and the increase in 1994 indicate a transition period with lowest resilience between the two regimes (regime I, 1974–1989 and regime II 1994–2006). Using our resilience index – *R* as an index for ecosystem state and relating it to pressure indices using PC1_force_ shows the shift between the two regimes and the transition period (Fig. 7). Between 1974 and 1988 the *R* suggests a higher resilience, but with the change in the species interaction and the multiple pressures, changing via a transition period into another regime. The years 1992 and 1993 were characterised by a low *R*, even though pressure was decreasing. After 1993, *R* increased again, but not to the initial level, staying constant even with a change in the pressure until about 2000 after which it started to decline again.

## Discussion

This study demonstrated that i) the regime shift in the Baltic Sea in the late 1980s is well reflected by the ENA indices, and ii) two different ecosystem regimes can be distinguished. The first regime between 1974 and 1988 reflected a more mature and balanced ecosystem, with more diverse flow structure, higher resilience, characterised also by high primary production, and high fishing pressure at relatively high trophic levels. The second regime, between 1994 and 2006, was characterised as a more stressed, less resilient regime with high primary production and high fishing pressure on lower TL species, indicating a more productive and linearized food-web. We hypothesise that the regime shift was caused by the interplay of multiple drivers: climate, eutrophication and fishing.

The macro descriptors (*A/C, H, MPL*) and food-web indices (*PCI, FCI, PFD*) indicate that after the regime shift (mid-1990) the ecosystem was more disturbed, more stressed, had an inverted maturation process, and experienced greater system flows (*TST*). Structure related indices (*A/C, H, AMI, MPL, H*) show that the internal structure of the food-web changed from a web-like to a more chain-like structure (see Fig. 3), where fewer groups were involved in the transfer of energy, and flows were constrained - channelling energy through specific pathways. Recycling related indices (*FCI, PFD*) show that after the second regime shift the ecosystem conserved less nutrients, needed more time to recover and channelled more matter directly into detritus due to less macrozoobenthos involvement in flows and increased phytoplankton biomass. Moreover, the increase in system recycling without detritus (*PCI*) shows short and fast cycles. This was also visible in the ecosystem turnover rate and *Q* index, which is due to the higher proportion of biomass of small, fast growing organisms in the food-web (i.e. sprat and small copepods) after the mid-1990s.

The changes in fisheries indicators during the first regime could be explained by a higher percentage of cod in the catches, and low *PPR/PP*, due to higher support of cod biomass from the detrital food-chain [39]. The second regime was characterised by increased sprat catches and the redirection of flows from benthic to pelagic pathways [39]. According to Tudela *et al*. [65], *PPR/PP* in combination with *mTLc* could be treated as an ecosystem index to capture the effect of fisheries and define “Ecosystem Overfishing”. After the regime shift there was an increase in *PPR/PP* and a drastic shift to lower *mTLc*. Therefore, this together with the trends/shift described above suggest ecosystem overfishing in the Baltic Sea.

A transition period, defined as the time when the ecosystem changed from one regime to another, was suggested by the low *R* between 1989 and 1993. It was characterized by high fluctuations of ecosystem structure (*AMI*, Fig. 3) and flows (*PCI*, *FCI*, *ToTP/TotB or TPP/TR,* Fig. 3), with the lowest system resilience (*R*, Fig. 7) during the studied time period. The transition occurred where the ecosystem was under high, constant pressure from eutrophication and cod and herring fisheries (Fig. 7). However, at the same time there was a stochastic overlap of hydrodynamic drivers, i.e., temperature, low cod reproductive volume (CodRV), probably affecting the change (Fig. S2 in File S1, see also Lindegren *et al*. [63]). After the transition period our analysis suggests that the system did not return to the initial regime when the external forcing was reduced.

Our ENA analysis suggests further that the resilience of the second regime is lower than the first, and therefore another significant disturbance of the ecosystem may cause the system to move to another alternative regime [27] similar to what happened in the Black Sea [64]. At this stage we are not able to say if the current regime is reversible or how stable it is. However, since 2006 higher cod biomass seems to suggest a possible change towards the first state [65].

Our results of forcing (abiotic factors) are in line with Möllmann *et al.* [18], [37] and the ICES Working Group on Integrated Assessment of the Baltic Sea (WGIAB) [60]. It also agrees with that of Kenny *et al.* [66] who showed a drivers shift in the 1980s for the North Sea. The second shift, during the mid-1990s (Tab. 2), has also been described by Möllmann *et al.* [18], although a similar shift was not detected by Kenny *et al.* [66] for the North Sea. In the mid-1980s, the North Atlantic Oscillation (NAO) and Baltic Sea Index (BSI) shifted sharply from a negative to a positive phase, affecting the hydrodynamic conditions, i.e. temperature, salinity and oxygen conditions throughout the whole area [64]. These climate anomalies most probably induced the simultaneous regime shift observed in the North Sea and Baltic Sea between 1987 and 1988 [17], [63].

Despite the fact that the model reproduces shifts in given functional groups relatively well (see Fig. S1 in File S1), and that the integrated analyses (Tab. 2–3 and Fig. 4–5) compare well to the results in Möllmann *et al.* [18], we are aware of the limitations of our analyses, such as high cross-correlation, the lack of seasonality and natural noise, as well as the aggregated and simplified food-web structure.

### Method Discussion

Recent advances in network science have encouraged ecologists to study food-webs through network indices [14], [67], [68]. The estimations of species interactions often benefit the understanding of ecosystem response to perturbations [10], [69], but it must be kept in mind that the impact of network structure on community may differ between different interaction types [70]. Consequently, the ENA analysis depends strongly on model quality and structure. As explained by Abarca-Arenas and Ulanowicz [71] and Pinnegar *et al*. [72] the number of functional groups and model structure have an impact on the number of flows and system properties. This has to be taken into account when comparing our results to other system outputs and other Baltic Sea models. Ecopath with Ecosim [73] is a commonly used approach that has been broadly discussed. Plagányi and Butterworth [74], Aydin [75], Coll *et al*. [76] and Walters *et al.* [77] described the pros and cons of the methodology, which has been taken into account during model building, fitting and evaluation [39], [78]. Niiranen *et al.* [78] found that data uncertainties may translate to uncertainties in modelled trophic control and hence results. However in this study the model was well fitted for several trophic levels and we have confidence in the model and data [39], which represent changes in biomasses and ecosystem dynamics well (see Fig. S1 in File S1).

### Management Outlook

Our results have significant implications for the understanding of the dynamics of the ecosystem [40], [79] and adaptive management [80].

With regard to overall performance and robustness, ecosystem level indicators based on ENA and food-web analysis are informative on intermediate and long time-scales [4], [81], [82]. But they are also difficult to use in annual updates of integrated assessments and advice, and may be more difficult for stakeholders to understand [82]. Nevertheless, examples of operational use do exist, e.g. the Puget Sound Integrated Ecosystem Assessment [83]. In addition, using food-web models and the ENA approach to explore different management scenarios, through changing fishing mortality of different species, nutrient loads, and/or hydrodynamic condition, could enable optimal management to ensure restoration, increasing ecosystem resilience and guard against future surprises.

## Conclusions

Our study revealed that the cumulative nature of anthropogenic stressors, such as fishing and eutrophication, needs to be analysed in combination with large scale environmental drivers (climate), ecosystem characteristics and emergent properties. This encapsulates the holistic approach needed for ecosystem based management. This is the first study where an abrupt regime shift was demonstrated by using an index of resilience calculated from the ecological network analysis using an Ecopath with Ecosim model that described the system as a whole.

## Supporting Information

File S1 Figure S1, Model fit to observed data (dots are observations, solid line are model estimates). The input data (annual biomass - B and cathes - C) and model estimates are expressed as t/km^2^ of wet weight. Figure S2, Model forcing anomalies relative to the initial value in 1974, 1974–2006 (note different scale). Where SST_aug is sea surface temperature in August; TempWC_spring is 0–50 m temperature in spring, PP_BALTSEM represents primary production, hypoxic is the area that is hypoxic, CodRV - Cod Reproductive Volume, HER_rec is herring recruitments anomalies, FSmallCod and FAdCod are anomalies of fishing morality of small and adult cod, FJuvSprat and FAdSprat, FJuvHerr, FAdHerr represent fishing mortality changes for adult and juvenile clupeid species. Figure S3, Modelled biomass anomalies (note different scale) 1974–2006. Table S1, Basic input to current EwE model (biomass is in t/km^2^, P/B and Q/B are annual ratios of production and consumption to biomass, EE is ecotrophic efficiency (proportion), P/Q is the ratio of production to consumption, TL is trophic level and the catch is in t/km^2^/yr. Table S2, Diet (proportion) composition matrix of used EwE model. Table S3, Vulnerabilities parameters obtained after model fitting. Table S4, PCA (PC1 and PC2) loadings - for graphic representation see Figure 4A and 4C. Table S5, Indices and definitions used. Table S6, Cross-correlations between indices.
(DOCX)
