# Supplementary material for: Ecological Network Indicators of Ecosystem Status and Change in the Baltic Sea
Source: PLoS One. 2013 Oct 7;8(10):e75439. doi: 10.1371/journal.pone.0075439 (PMC3792121; doi:10.1371/journal.pone.0075439)
Supplement: File S1 — Figure S1, Model fit to observed data (dots are observations, solid line are model estimates). The input data (annual biomass - B and cathes - C) and model estimates are expressed as t/km2 of wet weight. Figure S2, Model forcing anomalies relative to the initial value in 1974, 1974–2006 (note different scale). Where SST_aug is sea surface temperature in August; TempWC_spring is 0–50 m temperature in spring, PP_BALTSEM represents primary production, hypoxic is the area that is hypoxic, CodRV - Cod Reproductive Volume, HER_rec is herring recruitments anomalies, FSmallCod and FAdCod are anomalies of fishing morality of small and adult cod, FJuvSprat and FAdSprat, FJuvHerr, FAdHerr represent fishing mortality changes for adult and juvenile clupeid species. Figure S3, Modelled biomass anomalies (note different scale) 1974–2006. Table S1, Basic input to current EwE model (biomass is in t/km2, P/B and Q/B are annual ratios of production and consumption to biomass, EE is ecotrophic efficiency (proportion), P/Q is the ratio of production to consumption, TL is trophic level and the catch is in t/km2/yr. Table S2, Diet (proportion) composition matrix of used EwE model. Table S3, Vulnerabilities parameters obtained after model fitting. Table S4, PCA (PC1 and PC2) loadings - for graphic representation see Figure 4A and 4C. Table S5, Indices and definitions used. Table S6, Cross-correlations between indices. (DOCX) [file pone.0075439.s001.docx]

Supplementary materials_S1

Ecological network indicators of ecosystem status and change in the Baltic Sea

Maciej T. Tomczak^1^, Johanna J. Heymans^2^, Johanna Yletyinen^3^, Susa Niiranen^4^, Saskia A. Otto^4^ and Thorsten Blenckner^4^

^1^ Baltic Sea Centre, Stockholm University, SE-106 91 Stockholm, Sweden

^2^ Scottish Association for Marine Science, Scottish Marine Institute, Dunbeg, Oban, PA371QA, United Kingdom

^3^ Nordic Centre for Research on Marine Ecosystems and Resources under Climate Change (NorMER), Stockholm Resilience Centre, Stockholm University, SE-106 91 Stockholm, Sweden

^4^ Stockholm Resilience Centre, Stockholm University, SE-106 91 Stockholm, Sweden


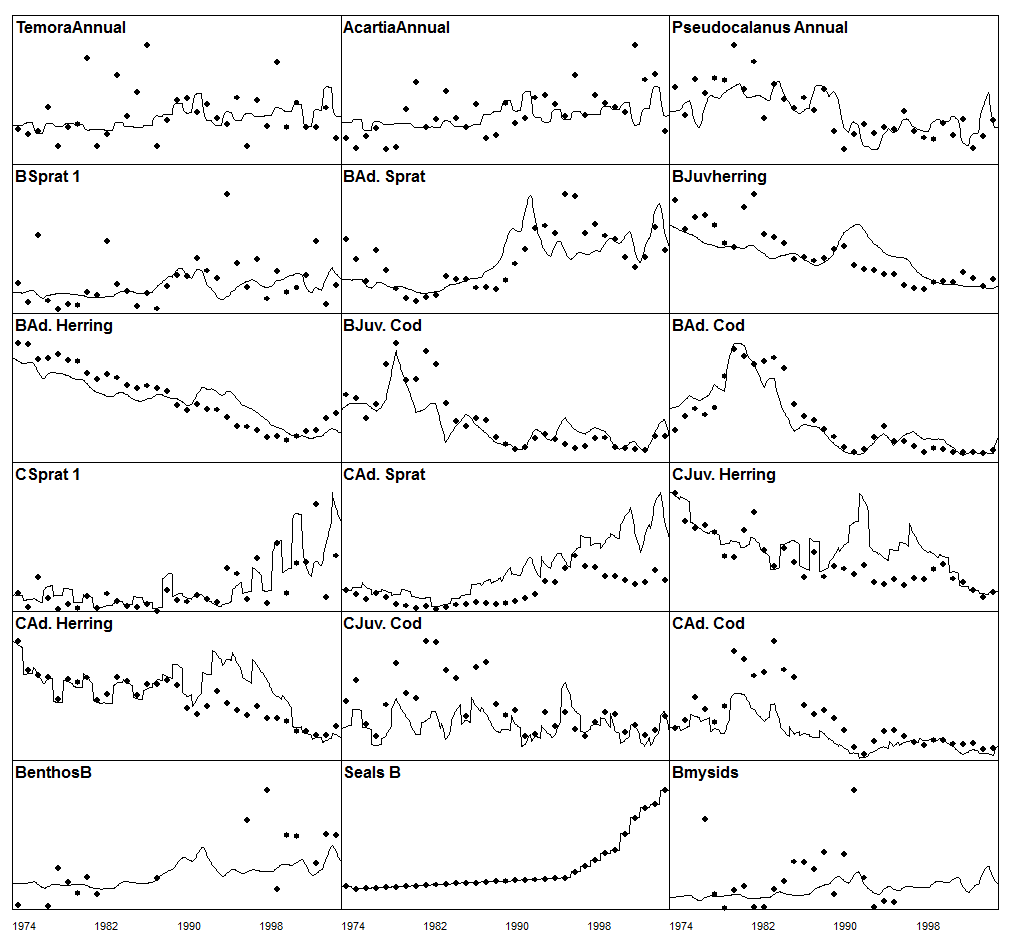


Figure S1. **Model fit to observed data** (dots are observations, solid line are model estimates). The input data (annual biomass - B and cathes - C) and model estimates are expressed as t/km^2^ of wet weight.


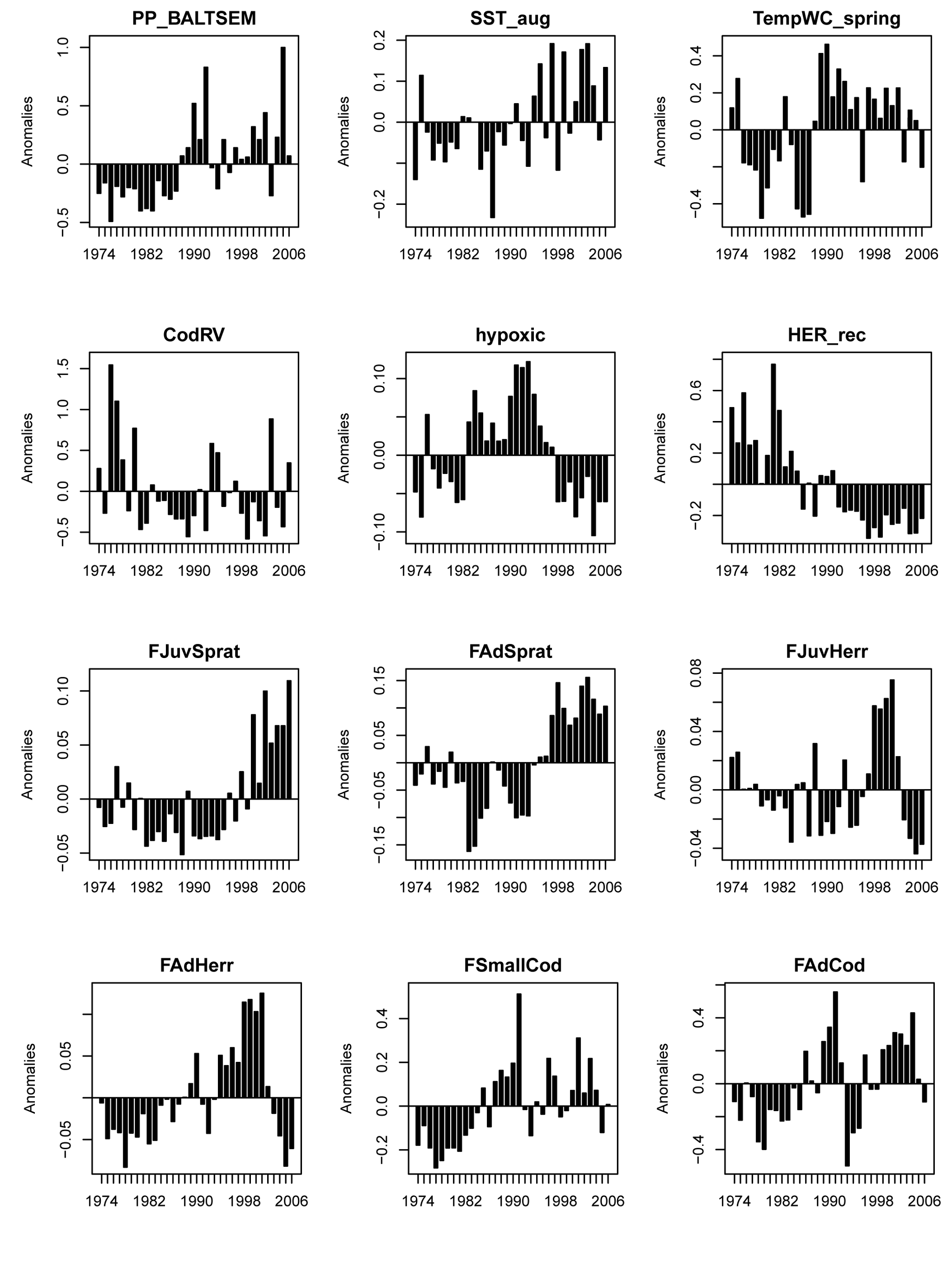
Figure S2. **Model forcing anomalies relative to the initial value in 1974, 1974-2006 (note different scale).** Where SST_aug is sea surface temperature in August; TempWC_spring is 0-50m temperature in spring, PP_BALTSEM represents primary production, hypoxic is the area that is hypoxic, CodRV - Cod Reproductive Volume, HER_rec is herring recruitments anomalies, FSmallCod and FAdCod are anomalies of fishing morality of small and adult cod, FJuvSprat and FAdSprat, FJuvHerr, FAdHerr represent fishing mortality changes for adult and juvenile clupeid species.


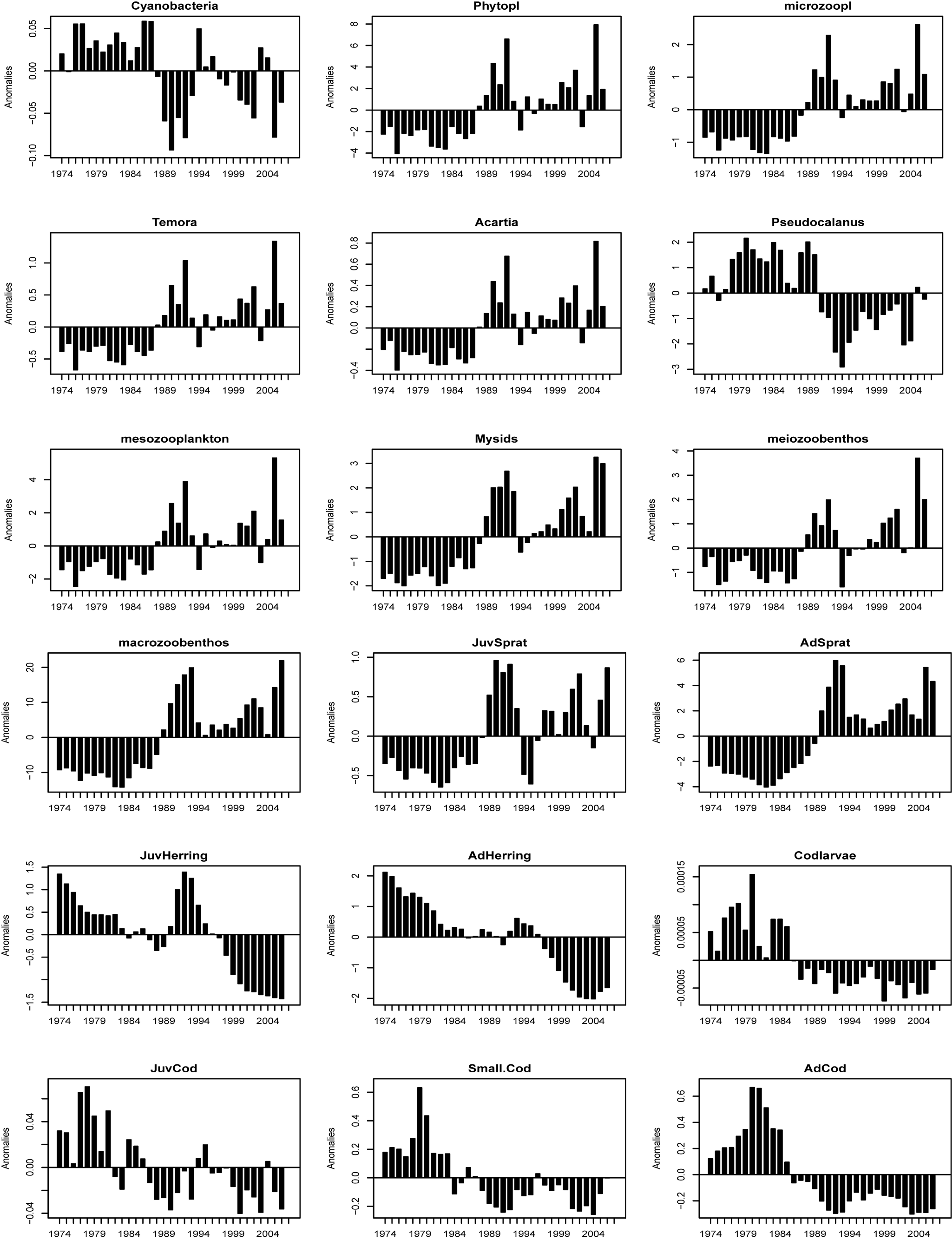


Figure S3. **Modelled biomass anomalies (note different scale) 1974-2006.**

Table S1. **Basic input to current EwE model** (biomass is in t/km^2^, P/B and Q/B are annual ratios of production and consumption to biomass, EE is ecotrophic efficiency (proportion), P/Q is the ratio of production to consumption, TL is trophic level and the catch is in t/km^2^/yr.

| **Group name** | **B** | **P/B** | **Q/B** | **EE** | **P/Q** | **TL** | **Catch** |
| --- | --- | --- | --- | --- | --- | --- | --- |
| Cyanobacteria | 3.4 | 160 |  | 0.057 |  | 1 |  |
| Phytoplankton | 7.3 | 135 |  | 0.827 |  | 1 |  |
| microzooplankton | 3.1 | 110 | 367 | 0.387 | 0.3 | 2 |  |
| Temora sp | 1.9 | 20 | 67 | 0.661 | 0.299 | 2.2 |  |
| Acartia sp | 1.35 | 20 | 67 | 0.55 | 0.299 | 2.2 |  |
| Pseudocalanus sp. | 4.3 | 8 | 27 | 0.538 | 0.296 | 2.3 |  |
| other mesozooplankton | 4 | 20 | 67 | 0.144 | 0.299 | 2.2 |  |
| Mysids | 2.7 | 5 | 20 | 0.216 | 0.25 | 2.5 |  |
| meiozoobenthos | 4.8 | 6.17 | 31.167 | 0.609 | 0.198 | 2 |  |
| macrozoobenthos | 25 | 1.92 | 11.1 | 0.222 | 0.173 | 2.1 |  |
| JuvSprat | 1.251 | 2 | 13.693 | 0.309 | 0.146 | 3.22 | 0.072 |
| AdSprat | 4.213 | 0.77 | 6.1 | 0.49 | 0.126 | 3.23 | 0.819 |
| JuvHerring | 4.049 | 2.2 | 5.159 | 0.2 | 0.426 | 3.28 | 0.53 |
| AdHerring | 5.619 | 0.42 | 2 | 0.619 | 0.21 | 3.3 | 1.01 |
| Cod larvae | 0.000154 | 0.5 | 75.89 | 0 | 0.007 | 3.28 | 0 |
| JuvCod | 0.0947 | 1.24 | 12.987 | 0.058 | 0.095 | 3.34 | 0 |
| Small Cod | 0.531 | 0.6 | 5.968 | 0.676 | 0.101 | 3.82 | 0.204 |
| AdCod | 0.49 | 1.04 | 3.96 | 0.733 | 0.263 | 4.01 | 0.373 |
| Seals | 0.0006 | 0.1 | 12.77 | 0 | 0.008 | 4.54 |  |
| Detritus-water column | 3255.81 |  |  | 0.877 |  | 1 |  |
| Detritus-sediment | 4651.17 |  |  | 0.665 |  | 1 |  |

Table S2. Diet (proportion) composition matrix of used EwE model.

| **No.** | **Prey \ Predator** | **3** | **4** | **5** | **6** | **7** | **8** | **9** | **10** | **11** | **12** | **13** | **14** | **15** | **16** | **17** | **18** | **19** |
| --- | --- | --- | --- | --- | --- | --- | --- | --- | --- | --- | --- | --- | --- | --- | --- | --- | --- | --- |
| 1 | Cyanobacteria | 0.001 | 0.05 | 0.05 | 0.05 | 0.05 |  |  |  |  |  |  |  |  |  |  |  |  |
| 2 | Phytoplankton | 0.45 | 0.55 | 0.4 | 0.4 | 0.5 | 0.3 |  |  |  |  |  |  |  |  |  |  |  |
| 3 | microzooplankton |  | 0.2 | 0.2 | 0.3 | 0.2 |  |  |  |  |  |  |  |  |  |  |  |  |
| 4 | Temora sp |  |  |  |  |  | 0.15 |  |  | 0.33 | 0.353 | 0.024 | 0.159 | 0.1 |  |  |  |  |
| 5 | Acartia sp |  |  |  |  |  | 0.1 |  |  | 0.3 | 0.138 | 0.014 | 0.043 | 0.1 |  |  |  |  |
| 6 | Pseudocalanus sp. |  |  |  |  |  | 0.05 |  |  | 0.199 | 0.307 | 0.023 | 0.356 | 0.8 |  |  |  |  |
| 7 | other mesozooplankton |  |  |  |  |  | 0.05 |  |  | 0.171 | 0.2 | 0.01 | 0.047 |  |  |  |  |  |
| 8 | Mysids |  |  |  |  |  |  |  |  | 0.0005 | 0.001 | 0.017 | 0.15 |  | 0.25 | 0.15 | 0.033 |  |
| 9 | meiozoobenthos |  |  |  |  |  |  |  | 0.065 |  |  |  |  |  |  |  |  |  |
| 10 | macrozoobenthos |  |  |  |  |  |  |  | 0.03 |  |  |  | 0.047 |  | 0.536 | 0.265 | 0.168 |  |
| 11 | JuvSprat |  |  |  |  |  |  |  |  |  |  |  | 0.003 |  | 0.008 | 0.151 | 0.093 |  |
| 12 | AdSprat |  |  |  |  |  |  |  |  |  |  |  |  |  | 0.037 | 0.136 | 0.151 | 0.042 |
| 13 | JuvHerring |  |  |  |  |  |  |  |  |  |  |  |  |  | 0.049 | 0.213 | 0.267 | 0.002 |
| 14 | AdHerring |  |  |  |  |  |  |  |  |  |  |  |  |  | 0.006 | 0.051 | 0.143 | 0.29 |
| 15 | Cod larvae |  |  |  |  |  |  |  |  |  |  |  |  |  |  |  |  |  |
| 16 | JuvCod |  |  |  |  |  |  |  |  |  |  |  |  |  |  | 0.002 | 0.001 | 0.011 |
| 17 | Small Cod |  |  |  |  |  |  |  |  |  |  |  |  |  |  |  | 0.005 | 0.179 |
| 18 | AdCod |  |  |  |  |  |  |  |  |  |  |  |  |  |  |  |  | 0.077 |
| 19 | Seals |  |  |  |  |  |  |  |  |  |  |  |  |  |  |  |  |  |
| 20 | Detritus-water column | 0.549 | 0.2 | 0.35 | 0.25 | 0.25 |  |  |  |  |  |  |  |  |  |  |  |  |
| 21 | Detritus-sediment |  |  |  |  |  | 0.2 | 1 | 0.905 |  |  |  |  |  |  |  |  |  |
| 22 | Import |  |  |  |  |  | 0.15 |  |  |  | 0.001 | 0.912 | 0.195 |  | 0.114 | 0.032 | 0.139 | 0.399 |
|  | Sum | 1 | 1 | 1 | 1 | 1 | 1 | 1 | 1 | 1 | 1 | 1 | 1 | 1 | 1 | 1 | 1 | 1 |

Table S3. Vulnerabilities parameters obtained after model fitting.

| **No.** | **Prey \ predator** | **3** | **4** | **5** | **6** | **7** | **8** | **9** | **10** | **11** | **12** | **13** | **14** | **15** | **16** | **17** | **18** | **19** |
| --- | --- | --- | --- | --- | --- | --- | --- | --- | --- | --- | --- | --- | --- | --- | --- | --- | --- | --- |
| 1 | Cyanobacteria | 2 | 4.12 | 1 | 2 | 2 |  |  |  |  |  |  |  |  |  |  |  |  |
| 2 | Phytoplankton | 2 | 1 | 1.02 | 2 | 2 | 2 |  |  |  |  |  |  |  |  |  |  |  |
| 3 | microzooplankton | | 1 | 1 | 2 | 2 |  |  |  |  |  |  |  |  |  |  |  |  |
| 4 | Temora sp |  |  |  |  |  | 2 |  |  | 1 | 1 | 2 | <100 | 2 |  |  |  |  |
| 5 | Acartia sp |  |  |  |  |  | 2 |  |  | 1 | 1 | 2 | 2 | 2 |  |  |  |  |
| 6 | Pseudocalanus sp. | |  |  |  |  | 2 |  |  | 5.64 | 6.79 | 2 | 2 | 2 |  |  |  |  |
| 7 | other mesozooplankton | | |  |  |  | 2 |  |  | 2 | 2 | 2 | 2 |  |  |  |  |  |
| 8 | Mysids |  |  |  |  |  |  |  |  | 2 | 2 | 2 | 2 |  | 2 | 2 | 2 |  |
| 9 | meiozoobenthos | |  |  |  |  |  |  | 2 |  |  |  |  |  |  |  |  |  |
| 10 | macrozoobenthos | |  |  |  |  |  |  | 2 |  |  |  | 2 |  | 2 | <100 | <100 |  |
| 11 | JuvSprat |  |  |  |  |  |  |  |  |  |  |  | 2 |  | 2 | 2 | <100 |  |
| 12 | AdSprat |  |  |  |  |  |  |  |  |  |  |  |  |  | 2 | 2 | <100 | 2 |
| 13 | JuvHerring |  |  |  |  |  |  |  |  |  |  |  |  |  | 2 | 1 | 2 | 2 |
| 14 | AdHerring |  |  |  |  |  |  |  |  |  |  |  |  |  | 2 | 2 | 2 | 2 |
| 15 | Cod larvae |  |  |  |  |  |  |  |  |  |  |  |  |  |  |  |  |  |
| 16 | JuvCod |  |  |  |  |  |  |  |  |  |  |  |  |  |  | 2 | 2 | 2 |
| 17 | Small Cod |  |  |  |  |  |  |  |  |  |  |  |  |  |  |  | 2 | 2 |
| 18 | AdCod |  |  |  |  |  |  |  |  |  |  |  |  |  |  |  |  | 2 |
| 19 | Seals |  |  |  |  |  |  |  |  |  |  |  |  |  |  |  |  |  |
| 20 | Detritus-water column | 2 | 1 | 1 | 2 | 2 |  |  |  |  |  |  |  |  |  |  |  |  |
| 21 | Detritus-sediment | |  |  |  |  | 2 | 2 | 2 |  |  |  |  |  |  |  |  |  |

Table S4. PCA (PC1 and PC2) loadings - for graphic representation see Figure 4A and 4C.

| **Variables** | **PC1** | **PC2** |
| --- | --- | --- |
| **Model Forcing*** |  |  |
| PP | 0.8775 | -0.39297 |
| SST_aug | 0.72 | 0.08582 |
| TempWC_spring | 0.5399 | -0.60051 |
| CodRV | -0.4749 | 0.44622 |
| Hypoxic Area | -0.5078 | -0.86499 |
| HER_rec | -0.9595 | 0.18102 |
| FJuvSprat | 0.8312 | 0.75249 |
| FAdSprat | 0.9322 | 0.59535 |
| FJuvHerr | 0.3018 | -0.13562 |
| FAdHerr | 0.598 | -0.62397 |
| FSmallCod | 0.7067 | -0.70581 |
| FAdCod | 0.8006 | -0.34479 |
| SealsBiomas | 0.9579 | 0.58677 |
|  |  |  |
| **Modeled Biomass** | **PC1** | **PC2** |
| Phytopl | 1.1073 | 0.3534 |
| microzoopl | 1.152 | 0.18686 |
| Temora | 1.1088 | 0.34977 |
| Acartia | 1.1041 | 0.34396 |
| Pseudocala | -0.5494 | 0.77701 |
| mesozoopl | 1.08 | 0.44504 |
| Mysids | 1.1552 | 0.0784 |
| meiozoo | 1.0394 | 0.47327 |
| macrozoo | 1.1161 | -0.09505 |
| Juvsprat | 1.0473 | 0.21894 |
| Adsprat | 1.125 | -0.12982 |
| JuvHerr | -0.6629 | 0.37951 |
| AdHerr | -0.8686 | 0.44596 |
| Codlar | -0.9412 | 0.4289 |
| Juvcod | -0.8239 | 0.31357 |
| SmallCod | -0.9364 | 0.33767 |
| AdCod | -1.0505 | 0.35261 |

Table S5. Indices and definitions used.

| **Indices** | **Formula** | **Definition** |
| --- | --- | --- |
| *TST* | $TST=\sum_{ij} T_{ij}$ | The total system throughput is defined as the sum of all flows (*T_ij_* is the flow between two compartments) in a particular ecosystem. It represents the “size of the entire system in terms of flow” [9] and its value is expected to decrease when a system becomes more degraded [21]. |
| *C* | $C=\sum_{ij} T_{ij}\log\left(\frac{T_{ij}}{TST}\right)$ | The development capacity (*C*) is a measure of potential of an ecosystem to develop [21,48] and the theoretical maximum of the ascendency (*A*) [9]. |
| *A* | $A=\sum_{i,j} (T_{ij})\cdot\log\left(\frac{T_{ij}\cdot TST}{T_{j}\cdot T_{i}}\right)$ | The ascendency (*A*) is defined as where *T_ij_* is the flow between two compartments and it includes all outflows from each compartment, *T_i_* is the sum of all material leaving the ith compartment, and *T_j_* is the sum of all flows entering the jth compartment [9]. *A* describes the growth and development of the system and it increases as a system matures [9]. |
| *A/C* | A/C | Relative ascendency *A/C* is the fraction of a potential food-web organization that is actually realized [9] and it is negatively related to maturity [43]. |
| *R* | $R=-\sum_{i=1}^{n} \sum_{j=1}^{n} (T_{ij})\cdot\log\left(\frac{T_{ij}^{2}}{\sum_{j=1}^{n} T_{ij}\cdot\sum_{i=1}^{n} T_{ij}}\right)$ | The Redundancy (*R*) [46]: indicates the system’s energy in reserve [49]. *R* is the best indicator of a change in the degrees of freedom of the system, and describes the distribution of energy flow among the ecosystem pathways [7]. Based on the description of *R* by Ulanowicz [46], who suggested that “it strongly ties to the effective multiplicity of parallel flows by which medium passes between any two arbitrary system components”, Heymans *et al.* [7] proposed *R* as an index of system resilience. |
| *AMI* | $AMI=\sum_{i,j} \left(\frac{T_{ij}}{TST}\right)*\log\left(\frac{T_{ij}*TST}{T_{j}*T_{i}}\right)$ | Average Mutual Information (*AMI*) measures the organization of the exchanges among components. *A* rise in *AMI* signifies that the system is becoming more constrained and is channelling flows along more specific pathways. [45]. *T_i_* is the sum of all material leaving the ith component and *T_j_* is the sum of all flows entering the jth component [46]. |
| *H* | $H=-\sum_{ij} \frac{T_{ij}}{TST}*\log\left(\frac{T_{ij}}{TST}\right)$ | Entrophy - by Ulanowicz [46], the diversity of flows or systems entropy (*H*) is an indication of the total uncertainty embodied in the given configuration of flows of the system, and represents the total number and diversity of flows in a system [25]. |
| *MPL* | *MPL* = throughput/sum of exports + respiration | The mean path length (*MPL*), accounts for the number of functional groups involved in a flow of matter [52] and represents the average number of groups that an inflow or outflow passes through [42]. The *MPL* is expected to decrease with fishing [21]. |
| *Q index* | $Q90=\frac{0.8S}{log(\frac{R_{2}}{R_{1}})}$ | Where S is the total number of functional groups in the model; R1 and R2 are the representative biomass values of the 10th and 90th percentiles in the cumulative abundance distribution.  *Kempton Q index* - The Q-90 statistic, a variant on Kempton's Q index, is developed to measure the effects of mortality from fishing or climate on species diversity in whole ecosystem simulation models that group functionally similar organisms. The statistic represents the slope of the cumulative species abundance curve between the 10- and 90-percentiles [47]. |
| *FCI* | $FCI=\frac{{TST}_{c}}{{TST}_{tot}}$ | The Finn’s cycling index (*FCI* [52]) is the proportion of the total system throughput (*TST*) that is recycled in the system. According to Monaco and Ulanowicz [49], cycling is considered to be an important indicator of an ecosystem’s ability to maintain its structure and integrity through positive feedback and is used as an indicator of stress [9] and systems maturity [43,50]. *FCI* is an indicator of the recovery time of an ecosystem through development of routes to conserve nutrients [50]. An increase in the *FCI* would mean the system would recover faster from a perturbation, whereas a system would be expected to take longer to recover (lower *FCI*) when it is in a more degraded state. |
| *PCI* | $PCI=\frac{{TST}_{no det}}{{TST}_{tot}}$ | The Predatory Cycling Index (*PCI*) - is a slightly modified *FCI*. *PCI* is calculated by excluding the cycling through detritus. Disturbed systems are characterized by short and fast cycles while complex trophic structures have long and slow recycling of matter [24,43,48]. |
| *PFD* | Proportion of total trophic flows that flow into the detritus box (t/km²/year). | The proportional flow to detritus (*PFD*) - it has been proposed that as fishing impact increases, this indicator increases due to disruption of energy paths in the food web [21]. |
| *ToTP/ToTB* | The Total Production / Total Biomass ratio | System turnover rate is an indicator of the average size of organisms in an ecosystem [21]. It is hypothesized that fishing reduces the mean size of organisms in ecosystems and it is expected to increase with fishing [21]. |
| *TPP/TR* | Total primary productivity / Total system respiration | The ratio between total primary productivity and total system respiration (*TPP/TR*) was proposed by Odum [54], indicating maturity/balanced of an ecosystem. Ratios close to 1 indicate mature/balanced ecosystems. |
| *PPR* | $PPR=\sum_{paths} \left[\frac{Y_{i}}{P_{i}}\cdot\prod_{j,i} \frac{Q_{j}}{P_{j}\times EE_{j}}\cdot DC_{j,i}\right]$ | Primary Production Required (*PPR*) - the primary production and detritus flows from TL 1 that are required to sustain fisheries (expressed as t/km²/year). This allows the evaluation and comparison of fishing activities across ecosystems. The *PPR* is obtained by calculating the flows backwards, expressed in primary production and detritus equivalents, for all pathways from the caught species down to the primary producers and detritus [38,51]. The *PPR* increases with fishing intensity. Y_i_ is the catch of a given group i, P_j_ is the production of predator j, Q_j_ is the consumption of predator j, DC_j_,i is the diet composition of each predator j/prey i interaction in each path and EE_j_ is the ecotrophic efficiency for compartment j, or the proportion of the production that is used within the system due to consumption or is exported from the system (e.g. in terms of catches). *PPR* was analysed also in reference to PP, to reflect a percentage of PP used to sustain catches. |
| *mTLc* | $mTLc=\frac{\sum_{j} Y_{j}\cdot TL_{j}}{\sum_{j} Y_{j}}$ | The Mean Trophic Level of Catch (*mTLc*) - Where *mTLc* is the mean trophic level of catch, TL_j_ - Trophic level of the caught species by their proportion in total landings (Y_j_/∑Y_j_) instead of the catches. TL of catch captures ‘fishing down marine food webs’ (Pauly et al., 1998) as removal of top predatory fish results in catches dominated by small, lower TL species. It is expected to decrease with fishing [21]. |
| *Total Catch* | ΣYj | Sum of all fishery catches extracted from modelled ecosystem at given year. |

| **Var** | ***TST*** | ***A/C*** | ***R*** | ***AMI*** | ***H*** | ***PCI*** | ***FCI*** | ***MPL*** | ***PFD*** | ***Biomass*** | ***ToTP/TotB*** | ***TPP/TR*** | ***Tot C*** | ***PPR/PP*** | ***DRc*** | ***mTLc*** | ***KempQ*** |
| --- | --- | --- | --- | --- | --- | --- | --- | --- | --- | --- | --- | --- | --- | --- | --- | --- | --- |
| *TST* | 1 | 0.22 | -0.94 | -0.65 | -0.96 | 0.25 | -0.98 | -0.96 | 0.86 | 0.99 | 0.61 | -0.21 | 0.69 | 0.36 | 0.35 | -0.73 | -0.84 |
| *A/C* | 0.22 | 1 | -0.51 | 0.57 | -0.24 | 0.95 | -0.16 | -0.29 | 0.63 | 0.27 | -0.47 | -0.88 | 0.44 | 0.58 | 0.63 | -0.56 | -0.4 |
| *R* | -0.94 | -0.51 | 1 | 0.37 | 0.9 | -0.54 | 0.93 | 0.96 | -0.98 | -0.95 | -0.37 | 0.49 | -0.77 | -0.58 | -0.57 | 0.84 | 0.88 |
| *AMI* | -0.65 | 0.57 | 0.37 | 1 | 0.66 | 0.54 | 0.68 | 0.56 | -0.2 | -0.59 | -0.93 | -0.59 | -0.22 | 0.2 | 0.27 | 0.22 | 0.43 |
| *H* | -0.96 | -0.24 | 0.9 | 0.66 | 1 | -0.22 | 0.94 | 0.93 | -0.8 | -0.95 | -0.67 | 0.1 | -0.66 | -0.29 | -0.26 | 0.77 | 0.88 |
| *PCI* | 0.25 | 0.95 | -0.54 | 0.54 | -0.22 | 1 | -0.2 | -0.33 | 0.68 | 0.32 | -0.53 | -0.94 | 0.34 | 0.64 | 0.69 | -0.48 | -0.44 |
| *FCI* | -0.98 | -0.16 | 0.93 | 0.68 | 0.94 | -0.2 | 1 | 0.99 | -0.85 | -0.97 | -0.66 | 0.15 | -0.7 | -0.41 | -0.37 | 0.76 | 0.87 |
| *MPL* | -0.96 | -0.29 | 0.96 | 0.56 | 0.93 | -0.33 | 0.99 | 1 | -0.91 | -0.96 | -0.56 | 0.28 | -0.74 | -0.5 | -0.46 | 0.81 | 0.9 |
| *PFD* | 0.86 | 0.63 | -0.98 | -0.2 | -0.8 | 0.68 | -0.85 | -0.91 | 1 | 0.89 | 0.19 | -0.64 | 0.72 | 0.66 | 0.66 | -0.82 | -0.86 |
| *Biomass* | 0.99 | 0.27 | -0.95 | -0.59 | -0.95 | 0.32 | -0.97 | -0.96 | 0.89 | 1 | 0.53 | -0.27 | 0.65 | 0.38 | 0.37 | -0.72 | -0.84 |
| *ToTP/TotB* | 0.61 | -0.47 | -0.37 | -0.93 | -0.67 | -0.53 | -0.66 | -0.56 | 0.19 | 0.53 | 1 | 0.59 | 0.41 | -0.1 | -0.18 | -0.37 | -0.47 |
| *TPP/TR* | -0.21 | -0.88 | 0.49 | -0.59 | 0.1 | -0.94 | 0.15 | 0.28 | -0.64 | -0.27 | 0.59 | 1 | -0.35 | -0.67 | -0.75 | 0.39 | 0.31 |
| *Tot C* | 0.69 | 0.44 | -0.77 | -0.22 | -0.66 | 0.34 | -0.7 | -0.74 | 0.72 | 0.65 | 0.41 | -0.35 | 1 | 0.57 | 0.54 | -0.84 | -0.66 |
| *PPR/PP* | 0.36 | 0.58 | -0.58 | 0.2 | -0.29 | 0.64 | -0.41 | -0.5 | 0.66 | 0.38 | -0.1 | -0.67 | 0.57 | 1 | 0.99 | -0.5 | -0.59 |
| *DRc* | 0.35 | 0.63 | -0.57 | 0.27 | -0.26 | 0.69 | -0.37 | -0.46 | 0.66 | 0.37 | -0.18 | -0.75 | 0.54 | 0.99 | 1 | -0.45 | -0.53 |
| *mTLc* | -0.73 | -0.56 | 0.84 | 0.22 | 0.77 | -0.48 | 0.76 | 0.81 | -0.82 | -0.72 | -0.37 | 0.39 | -0.84 | -0.5 | -0.45 | 1 | 0.8 |
| *KempQ* | -0.84 | -0.4 | 0.88 | 0.43 | 0.88 | -0.44 | 0.87 | 0.9 | -0.86 | -0.84 | -0.47 | 0.31 | -0.66 | -0.59 | -0.53 | 0.8 | 1 |

Table S6. Cross-correlations between indices
